# Anatomy and histology of the olfactory organ of the javelin goby *Synechogobius hasta* (Gobiiformes, Gobiidae)

**DOI:** 10.1186/s42649-024-00105-z

**Published:** 2024-12-17

**Authors:** Hyun-Tae Kim

**Affiliations:** Department of Science Education, Jeonju National University of Education, Jeonju, 55101 Republic of Korea

**Keywords:** Anatomy, Histology olfactory organ, Nostril, Nasal sac, Erythrocyte, *Synechogobius*, Tidal pool

## Abstract

The olfactory organ of *Synechogobius hasta* was investigated with a focus on its environmental adaptation, using stereo microscopy and light microscopy. This research revealed the following anatomical and histological characteristics: (i) tubular anterior nostril, (ii) one longitudinal lamella, (iii) two accessory nasal sacs, (iv) lymphatic cells in the lower part of the sensory epithelium, (v) four to five villi of olfactory receptor neurons, (vi) abundant blood capillaries beneath the sensory epithelium, and (vii) rod-shaped erythrocytes. These findings hint that the olfactory organ of *S*. *hasta* has anatomical and histological adaptations to intertidal pools that undergo periodic hypoxia and increased temperature under stagnant water conditions due to the tidal cycle.

## Introduction

Chemical signaling through olfaction in teleosts is crucial for various ecological behaviors, such as foraging, predator avoidance, reproduction, habitat detection, intraspecies recognition, and migration (Kasumyan 2004; Kiyokawa et al. 2013; Kermen et al. 2020). These behaviors have driven structural modifications in the position, number, and shape of olfactory tissues to adapt to the physical and chemical conditions of surrounding habitats (Zeiske et al. 1992; Kasumyan 2004). More specifically, intertidal fishes inhabiting estuaries or coastal areas exhibit unique adaptations to the variable conditions caused by tidal cycles, such as (i) tubular nostrils, (ii) elongated olfactory chambers (OC), (iii) accessory nasal sacs (ANS) with mucus, (iv) restricted distribution of sensory epithelium (SE), and (v) shorter dendrites on receptor neuron knobs (Belanger et al. 2003; Kim et al. 2019; Kim and Park 2020, 2021).

The javelin goby *Synechogobius hasta* is found along the coasts of Japan, China, Taiwan, and the western and southern seas of Korea, typically inhabiting muddy or sandy bottoms of estuaries, intertidal zones, and coastal areas (Kim and Park 2002). In these habitats, some air-breathing intertidal fishes have developed morphological and histological adaptations to survive in extreme conditions, such as low water volume, high water temperatures up to 40 °C, and partial hypoxia. Similarly, *S*. *hasta* has shown high viability in adverse environments like intertidal pools or valleys formed by tidal cycles, although it is not an air-breathing fish (Choi et al. 2005). The gobiid fishes share certain morphological and histological features adapted to the physical and chemical conditions of such tidal zones but differ in the details, such as nostril diameter, number of nasal sacs, lymphatic cell density, and ultrastructure of the sensory surface. As a result, variable modifications of their olfactory organs are considered unique adaptations to their microhabitats and ecological niches in intertidal zones and are used as taxonomic keys. However, the olfactory organ of *S*. *hasta* has not been studied in detail to understand its anatomical and histological adaptations. Therefore, the purpose of this study was to describe the olfactory organ of *S*. *hasta*, analyze its relationships to habitat conditions and ecology, and compare it with the olfactory organs of related species.

## Materials and methods

### Specimen collection

**Fig. 1:**
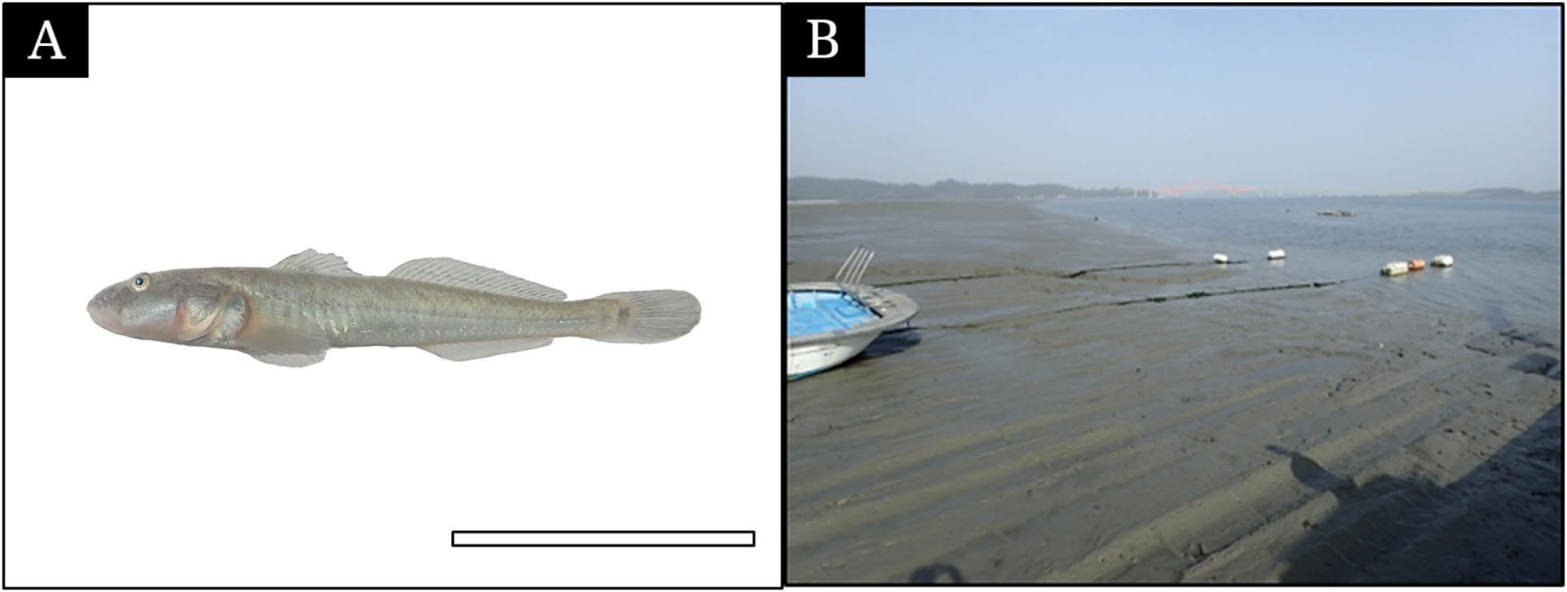
The photograph of *Synechogobius hasta* (**A**) and its intertidal habitat (**B**, N35°01’53” E126°08’59”). The bar indicates 10 cm

In May 2024, we collected 20 adult gobies (*Synechogobius hasta*, 74.3 to 113.7 mm standard length, Fig. 1A) using a casting net (5 × 5 mm mesh) from the intertidal zone of Dangchon-ri, Shinangun, Jeollanam-do, South Korea (Fig. 1B). After transporting the fish to the laboratory, we anesthetized them with MS-222 (Sigma, St Louis, MO, USA). Six fish were fixed in 10% buffered formalin solution (pH 7.4), and the rest were fixed in 2.5% buffered glutaraldehyde (GA) solution (pH 7.4). All procedures followed the guidelines of the Jeonbuk National University Institutional Animal Care and Use Committee.

### Microscopic investigation

We dissected and photographed the olfactory organs utilizing a surgical blade, a stereo microscope (SM; Stemi DV4, Carl Zeiss, Germany), and a digital camera (TG-3, Olympus, Japan). For light microscopy, the olfactory organs fixed in pH 7.4 buffered formalin were rinsed with tap water for 24 h. They were then processed through a graded alcohol series (70%, 80%, 90%, 95%, 100%), cleared with xylene, and embedded in paraffin wax. Sections of 5 μm thickness were cut from the paraffin blocks. The tissue sections were stained with hematoxylin and eosin (H&E) and Masson’s trichrome and observed under a light microscope (LM, Axio imager A2, Carl Zeiss, Germany). For scanning electron microscopy, the olfactory tissues preserved in pH 7.4 buffered 2.5% glutaraldehyde solution were rinsed three times with same buffer for 20 min each. Subsequently, they were post-fixed with 2% OsO_4_ in 0.1 M potassium phosphate buffer (pH 7.4), dehydrated through a graded alcohol series (50%, 60%, 70%, 80%, 90%, 95%, 100%), and immersed in tert-butyl alcohol. The olfactory organs were dried using a freeze-dryer (VFD-21 S, Vacuum Device Co., Ltd., Ibaragi, Japan), coated with OsO4 powder, and examined under a scanning electron microscope (SEM; SUPRA40VP, Carl Zeiss, Germany).

## Results

### Anatomy

**Fig. 2:**
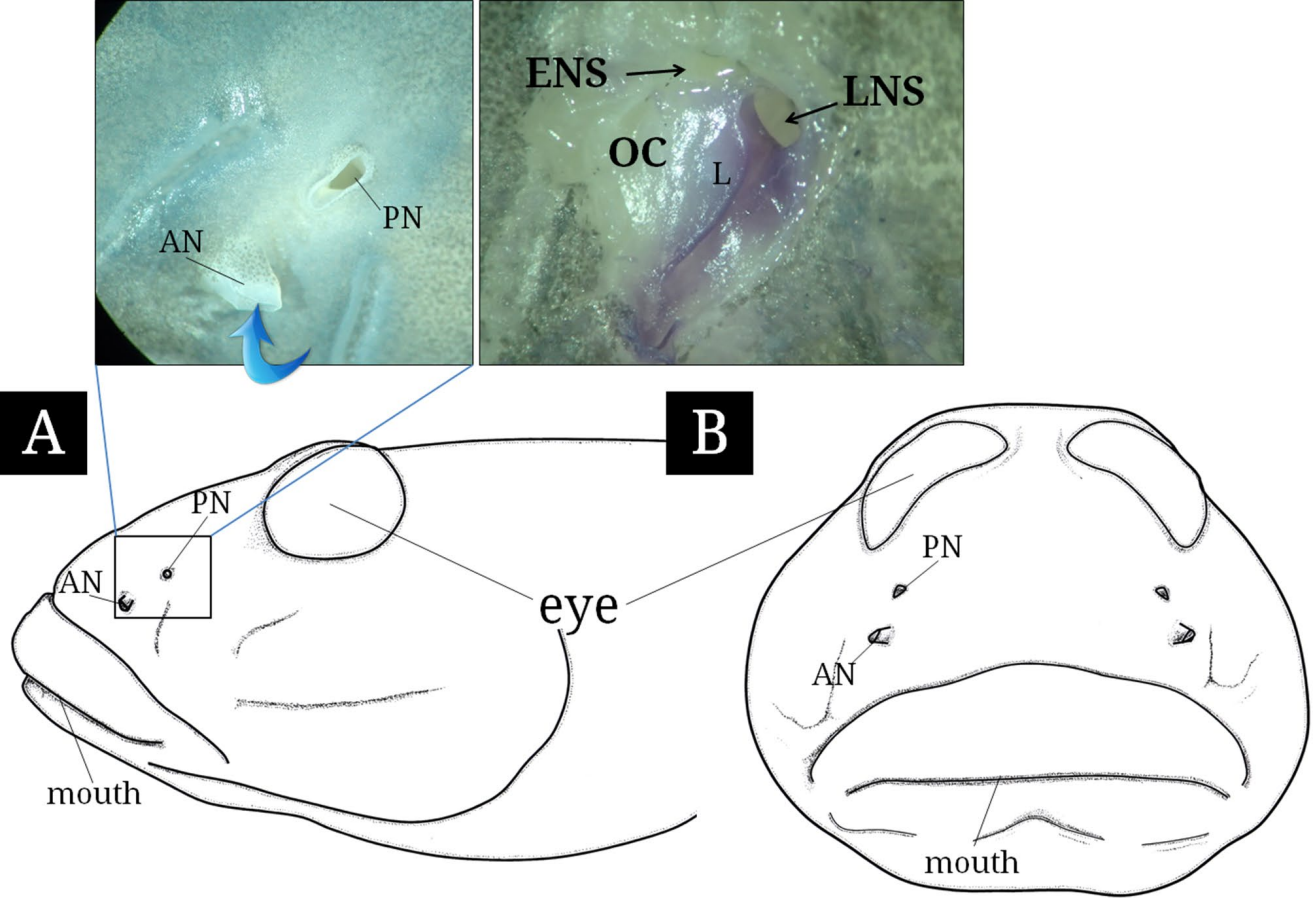
Schematic diagram of the lateral and front view of the head of *Synechogobius hasta* and the external (left photograph) and internal (right) structure of its olfactory organ. The blue arrow indicates water flowing. AN, anterior nostril; ENS, ethmoidal nasal sac; L, lamellae; LNS, lacrimal nasal sac; OC, olfactory chamber; PN, posterior nostril

The paired olfactory organs of *S. hasta* are located on the dorsal region of the snout (Fig. 2). Externally, they show two openings, the anterior (AN) and posterior nostrils (PN). The distance between these nostrils ranged from 1.37 to 1.58 mm. The AN, which measured between 0.50 and 0.72 mm in diameter, forms a tubular structure that protrudes from the skin surface, while the PN with a measured diameter of 0.42 to 0.70 mm is almost parallel to the skin surface. Internally, the OC contained only a single longitudinal lamella and two ANSs (ethmoidal and lacrimal sacs) extending internally toward the rear.

### Histology

**Fig. 3:**
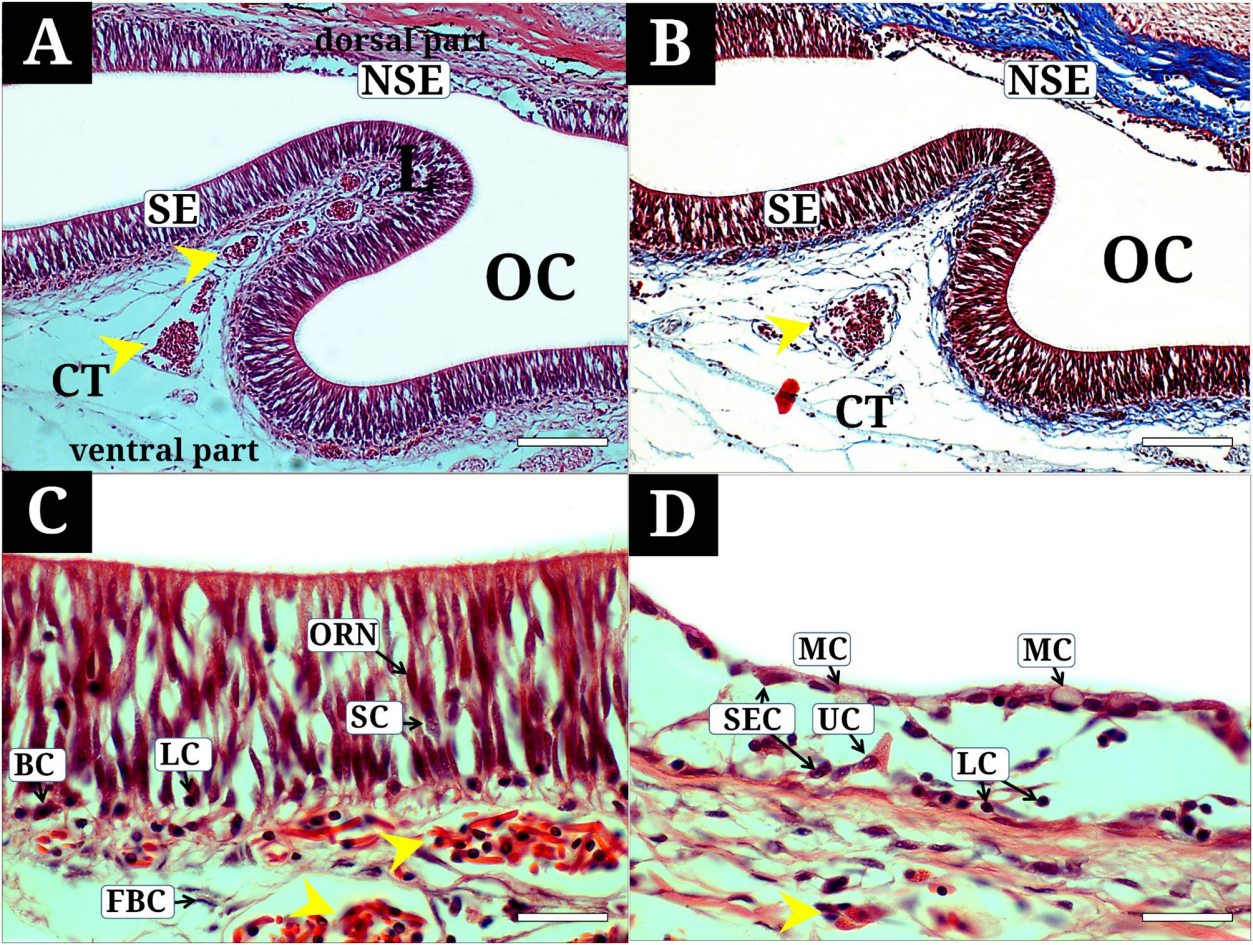
Histological characteristics of the olfactory epithelium of *Synechogobius hasta*, stained with hematoxylin and eosin (**A**, **C**, **D**), Masson’s trichrome (**B**). (**A**, **B**), the olfactory epithelium composed of sensory and non-sensory regions; (**C**) the sensory epithelium showing olfactory receptor neurons, supporting cells, basal cells, and lymphatic cells, and numerous blood capillaries and fibroblast cells in the connective tissue; (**D**) the non-sensory epithelium showing stratified epithelial cells, mucous cells, lymphatic cells, and unidentified cells. BC, basal cell; CT, connective tissue; FBC, fibroblast cell; L, lamella; LC, lymphatic cell; MC, mucous cell; NSE, non-sensory epithelium; OC, olfactory chamber; ORN, olfactory receptor neuron; SE, sensory epithelium; SEC, stratified epithelial cell; SC, supporting cells; unidentified cells, UC; yellow asterisk, blood capillary. The bars indicate 200 μm in A and B, 50 μm in C and D, respectively

The OC contains a single lamella arranged longitudinally, covered by olfactory epithelium (OE) that includes both SE and non-sensory regions (NSE) on each side (Fig. 3A and B). The SE is comprised of olfactory receptor neurons (ORN), supporting cells (SC), lymphatic cells (LC), and basal cells (BC) (Fig. 3C). ORNs are long bipolar neurons that extend from the base to the surface of the SE and are characterized by elongated dark brown nuclei stained with hematoxylin and eosin and Masson’s trichrome. SCs, which support the ORNs, are cylindrical with larger nuclei and were lighter brown compared to the ORNs in hematoxylin and eosin staining. LCs are the smallest circular cells with minimal cytoplasm and were stained dark purple with hematoxylin and eosin. BCs are oval-shaped cells located both vertically and horizontally along the basement membrane, with reddish-brown cytoplasm and deep purple nuclei when stained with hematoxylin and eosin. The NSE consists of mucous cells (MC), stratified epithelial cells (SEC), lymphatic cells (LC), and unidentified cells (UC) (Fig. 3D). MCs are large round cells with a flattened nucleus at the bottom and an unstained broad cytoplasm. Their nuclei stained dark brown with hematoxylin and eosin. SECs are polygonal cells that make up most of the NSE, with dark-brown-stained nuclei and faintly stained abundant cytoplasm. LCs similar to those of the SE are primarily located in the lower regions of the NSE. UCs are flask-shaped cells narrowing from the base toward the surface and featuring a prominent nucleus stained purple and weakly red-stained cytoplasm.

Numerous blood capillaries with prominent nuclei are found in the connective tissue beneath the SE (Fig. 3A-C), while they are sparse beneath the NSE (Fig. 3D). Most erythrocytes exhibit a rod-shaped structure. Fibroblast cells with flattened nuclei are observed throughout the connective tissue.

**Fig. 4:**
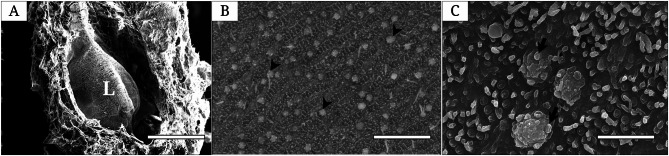
Scanning electron micrographs showing the olfactory lamella (**A**) and the surface of the sensory epithelium (**B** and **C**) of *Synechogobius hasta*. Bars indicate 500 *μ*m in A, 5 *μ*m in B, 500 nm in C. Arrow, olfactory receptor neuron’s dendrite; Arrowhead, olfactory receptor neuron; L, lamella

The single longitudinal lamella of the OC showed ORNs and numerous micro-protrusions (Fig. 4). ORNs had four to five villi on the olfactory knob as its dendrite on sensory surface (Fig. 4B and C). Numerous micro-protrusions were scattered on almost sensory surface (Fig. 4C).

## Discussion

The javelin goby *Synechogobius hasta* is typically found in coastal areas, but it also spends time in shallow tide pools for feeding and spawning during low tide in the intertidal zone. Tide pools throughout this zone generally experience stagnant water conditions until the next high tide, leading to low oxygen levels, increased water temperature, and exposure to air and UV radiation from the sun (Gosselin and Jones 2010; Richards 2011). Despite these harsh conditions, *S. hasta* demonstrates remarkable resilience and thrives in such environments (Choi et al. 2005). In the present study, *S*. *hasta* revealed the anatomical and histological characteristics of the olfactory organ related to the environmental conditions noted above as follows: (i) tubular anterior nostril, (ii) one longitudinal lamella, (iii) two accessory nasal sacs, (iv) LCs in the lower part of the SE, (v) four to five villi of the ORN, (vi) abundant blood capillaries beneath the SE, and (vii) rod-shaped erythrocytes.

The anterior nostrils of teleosts are closely associated with their swimming behavior and the water flow in their habitats (Atta 2013). Among teleosts, benthic or bentho-pelagic fishes living in water systems with little to no water flow typically exhibit tube-like anterior nostrils (Zeiske et al. 1992). This form has been confirmed in benthic gobies in intertidal zone, such as the round goby *Neogobius melanostomus* (Belanger et al. 2003), the eel goby *Odontamblyopus lacepedii* (Kim and Park 2018), the Kestrel goby *Gobius xoriguer* (Iglesias et al. 2021), and the limnetic goby *Redigobius fotuno* (Kobayashi et al. 2024). Tube-like nostrils are known to assist the olfactory chamber in creating the suction force for drawing in external water through conjunction and relaxation with skeletal movement of the head (Atta 2013; Kim et al. 2019). Therefore, this form of *S*. *hasta* is considered to have a crucial morphological adaptation for water intake in the shallow and stagnant water of intertidal pools.

The intertidal zones of South Korea exhibit regional differences in bottom particles, such as rocks, sand, and mud, depending on the latitude (Hwang et al. 2010). Even within the same region, the upper and lower parts of the intertidal zone, influenced by the tidal cycle, also show significant variations in water flow, current, and substrate composition (Chang and Choi 1998; De Boer 1998). With diverse habitats, the Family Gobiidae shows high numbers of species with unique ecological adaptations to niche intertidal zones (Jaafar and Murdy 2017). Among them, the amphibious mudskippers *Periophthalmus* and *Boleophthalmus*, which are well adapted to exposure to air on land, have no olfactory lamella, whereas most of the underwater Gobiidae species contain a single lamella within the olfactory chamber (Kuciel et al. 2013). While it is not clear that this characteristic is related to habitat or ecology, single longitudinal lamella such as in *S*. *hasta* may be useful for taxonomic classification of species.

As is found in many intertidal gobies, *S. hasta* showed two accessory nasal sacs, ethmoidal and lacrimal sacs. The number and shape of the accessory nasal sacs in gobiid fish vary by species as follows: *Scartelaos gigas* (Kim et al. 2014) and *Boleophthalmus pectinirostris* (Kim and Park 2020) possess two nasal sacs, and *Periophthalmus modestus* has one, whereas *Luciogobius guttatus* (Kim and Park 2016) and *Favonigobius gymnauchen* (Kim and Park 2016) are lacking nasal sacs. Functionally, Kim et al. (2019) noted that the accessory nasal sac provides direct suction force to facilitate the intake of external water into the olfactory chamber through its contraction and relaxation, especially when fish are in shallow and stagnant water. In addition, Aicardi et al. (2022) opined that a pumping mechanism in the accessory nasal sac facilitates water ventilation in the olfactory chamber with mouth and head movements. Therefore, the accessory nasal sacs of *S*. *hasta* may be considered to assist with the movement of water mixed with variable odor chemicals present in shallow and stagnant intertidal pools. In addition, the number and morphology of nasal sacs could be useful taxonomic characteristics, at least in the subfamily Oxudercidae.

Abundant blood capillaries were confirmed in the connective tissue beneath the SE of *S*. *hasta*. This could be considered a histological characteristic allowing large amounts of oxygen and carbon dioxide exchange in the SE of *S*. *hasta*. Moreover, most of the erythrocytes inside the capillary formed a rod-shaped structure. Research on the rod-shaped red blood cells in teleost fish is lacking. However, Rahman et al. (2019) documented that higher water temperature (31 ∼ 34 C) can cause morphological modification of red blood cells to interfere with the immune system of red spotted grouper. Andreyeva et al. (2017) confirmed that oxygen deficiency underwater leads to bidirectional changes in the volume of erythrocytes and their nuclei with a decrease in erythrocyte membrane permeability. This suggests that the morphology of *S*. *hasta* erythrocyte is related to hypoxia conditions or increased water temperature of the habitat, as in an intertidal pool. However, further research is needed to clarify the cause of the observed transformation in the erythrocyte morphology in response to environmental conditions.

These results might indicate that the olfactory organ of *S*. *hasta* has undergone anatomical and histological adaptations to intertidal conditions involving periodic hypoxia and increased water temperature of stagnant water due to the tidal cycle.

## Conclusions

The anatomical and histological characteristics of the olfactory organ of the javelin goby *Synechogobius hasta* were investigated with a focus on their adaptation to hypoxic habitats, which are formed in response to the periodic tidal cycle. The following microscopic findings were revealed: (i) tubular anterior nostril, (ii) one longitudinal lamella, (iii) two accessory nasal sacs, (iv) lymphatic cells in the lower part of the sensory epithelium, (v) four to five villi of olfactory receptor neurons, (vi) abundant blood capillaries beneath the SE, and (vii) rod-shaped erythrocytes. These results indicate anatomical and histological olfactory adaptations of fish to survive in adverse aquatic environments, such as tidal pools.

## Data Availability

Not applicable.

## Abbreviations

AN: Anterior nostril
ANS: Accessory nasal sac
BC: Basal cell
LC: Lymphatic cell
MC: Mucous cell
OC: Olfactory chamber
OE: Olfactory epithelium
ORN: Olfactory receptor neuron
PN: Posterior nostril
SC: Supporting cell
SE: Sensory epithelium
SEC: Stratified epithelial cell
UC: Unidentified cell
